# Supplementary material for: Target-agnostic identification of human antibodies to Plasmodium falciparum sexual forms reveals cross-stage recognition of glutamate-rich repeats
Source: eLife. 2025 Jan 16;13:RP97865. doi: 10.7554/eLife.97865 (PMC11737873; doi:10.7554/eLife.97865)
Supplement: Figure 2—source data 2. [file elife-97865-fig2-data2.zip › Figure 2C blots with labels.pptx]

## Slide 1
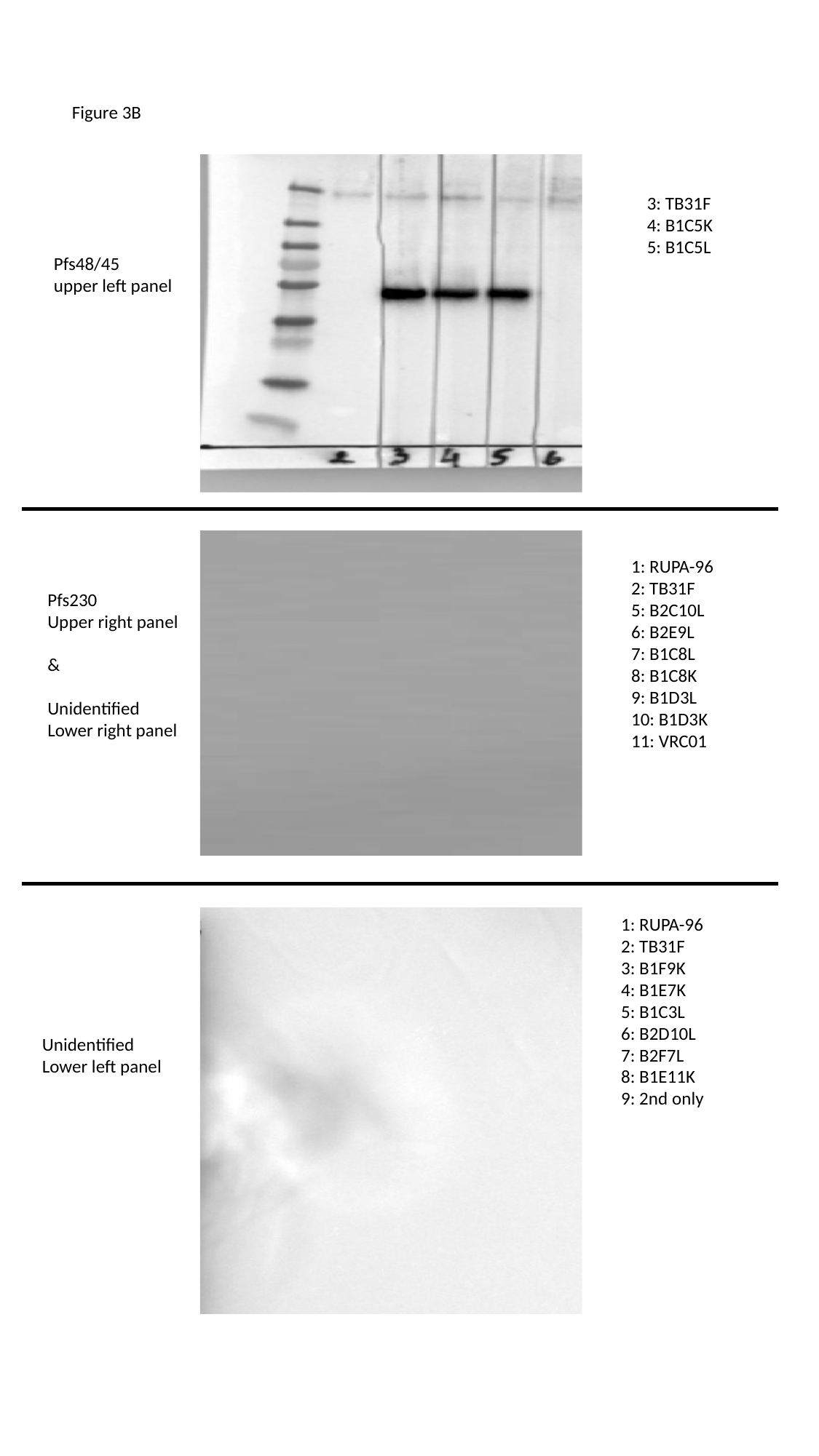

Figure 3B
3: TB31F
4: B1C5K
5: B1C5L
Pfs48/45
upper left panel
1: RUPA-96
2: TB31F
5: B2C10L
6: B2E9L
7: B1C8L
8: B1C8K
9: B1D3L
10: B1D3K
11: VRC01
Pfs230
Upper right panel
&
Unidentified
Lower right panel
1: RUPA-96
2: TB31F
3: B1F9K
4: B1E7K
5: B1C3L
6: B2D10L
7: B2F7L
8: B1E11K
9: 2nd only
Unidentified
Lower left panel
