# Supplementary figures and images for: Target-agnostic identification of human antibodies to Plasmodium falciparum sexual forms reveals cross-stage recognition of glutamate-rich repeats
Source: eLife. 2025 Jan 16;13:RP97865. doi: 10.7554/eLife.97865 (PMC11737873; doi:10.7554/eLife.97865)

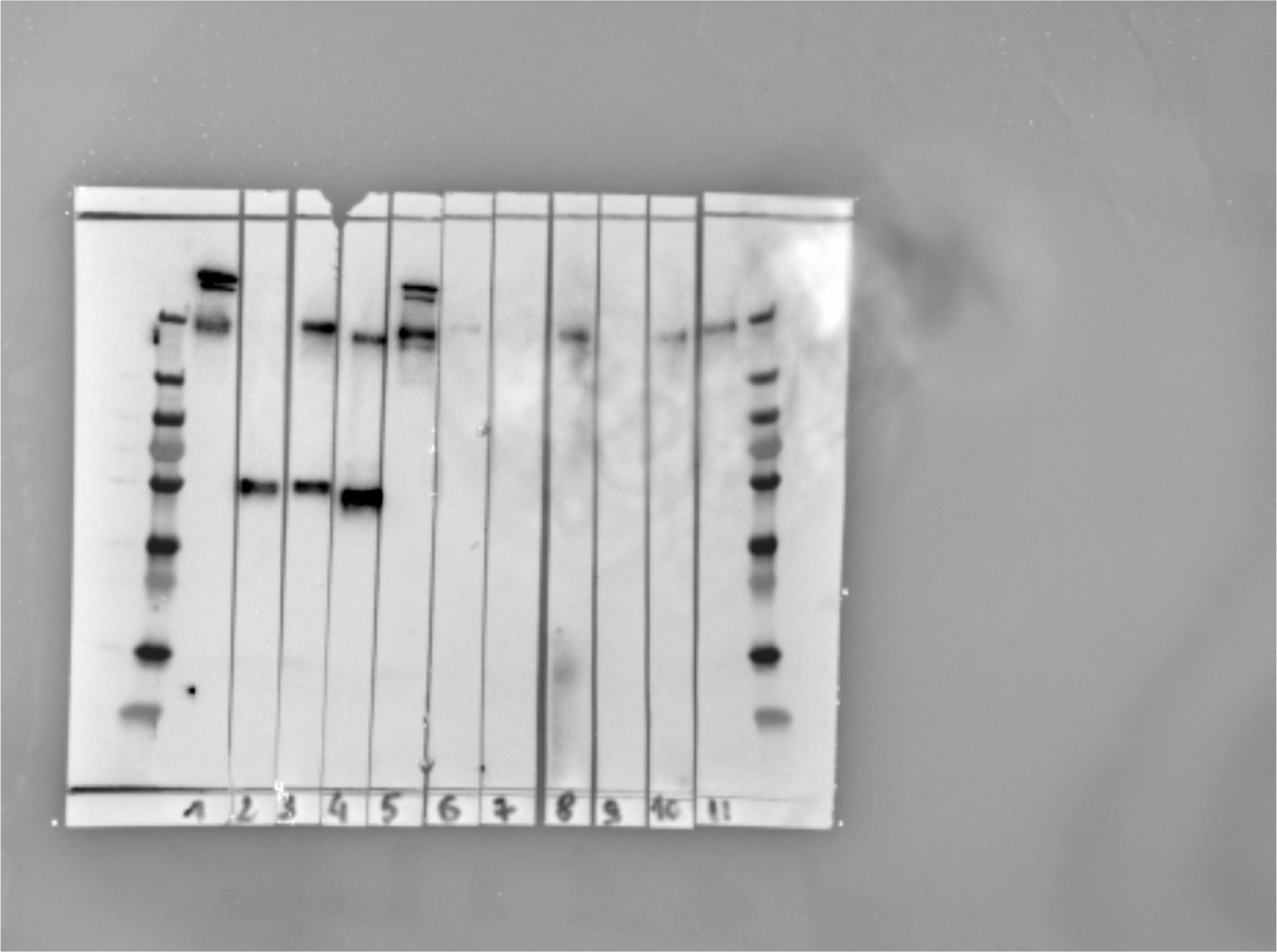

Supplement: Figure 2—source data 2. [file elife-97865-fig2-data2.zip › Figure 2C Pfs230 and unidentified antigens lower right panel.tif]

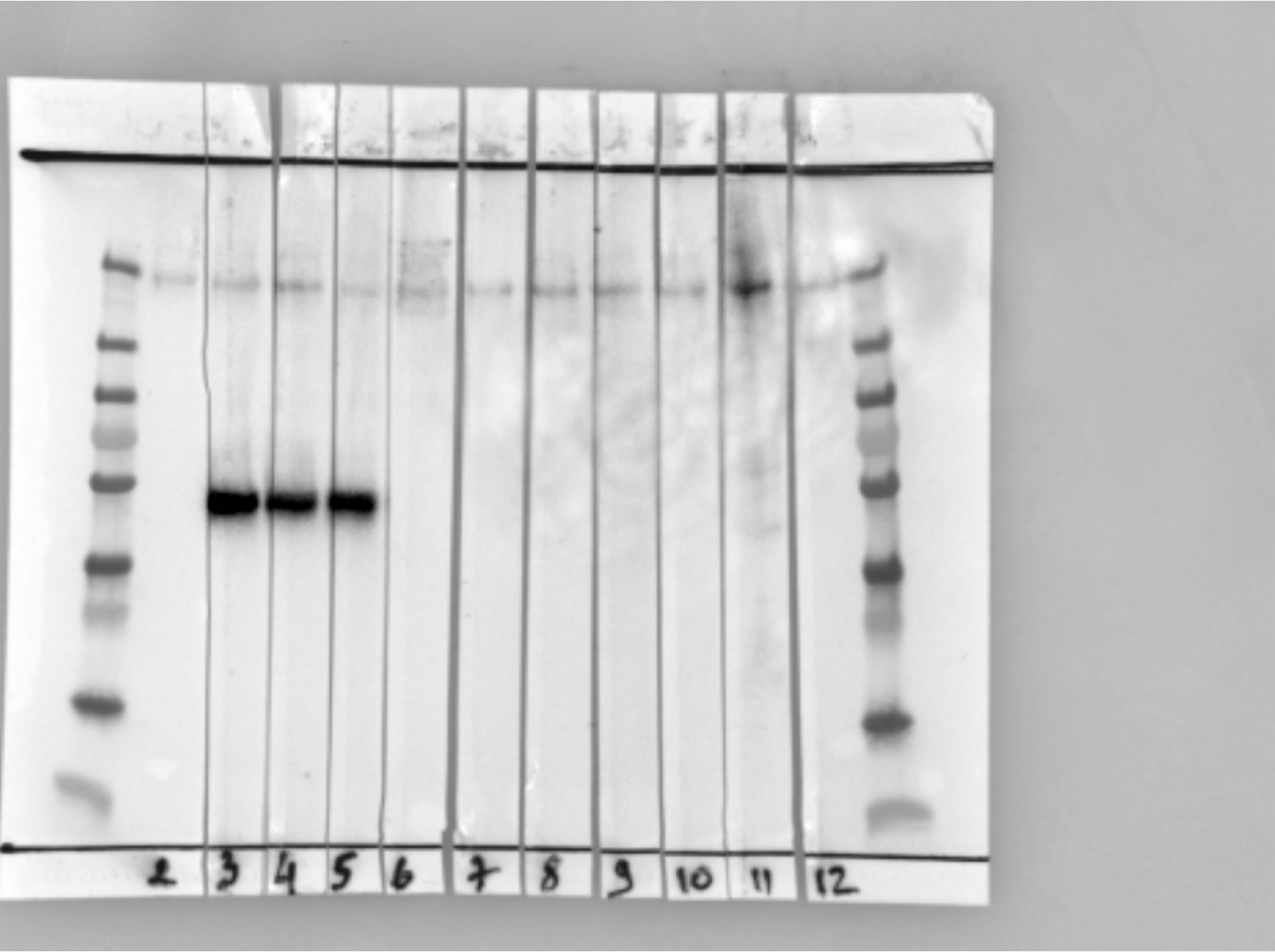

Supplement: Figure 2—source data 2. [file elife-97865-fig2-data2.zip › Figure 2C Pfs4845.tif]

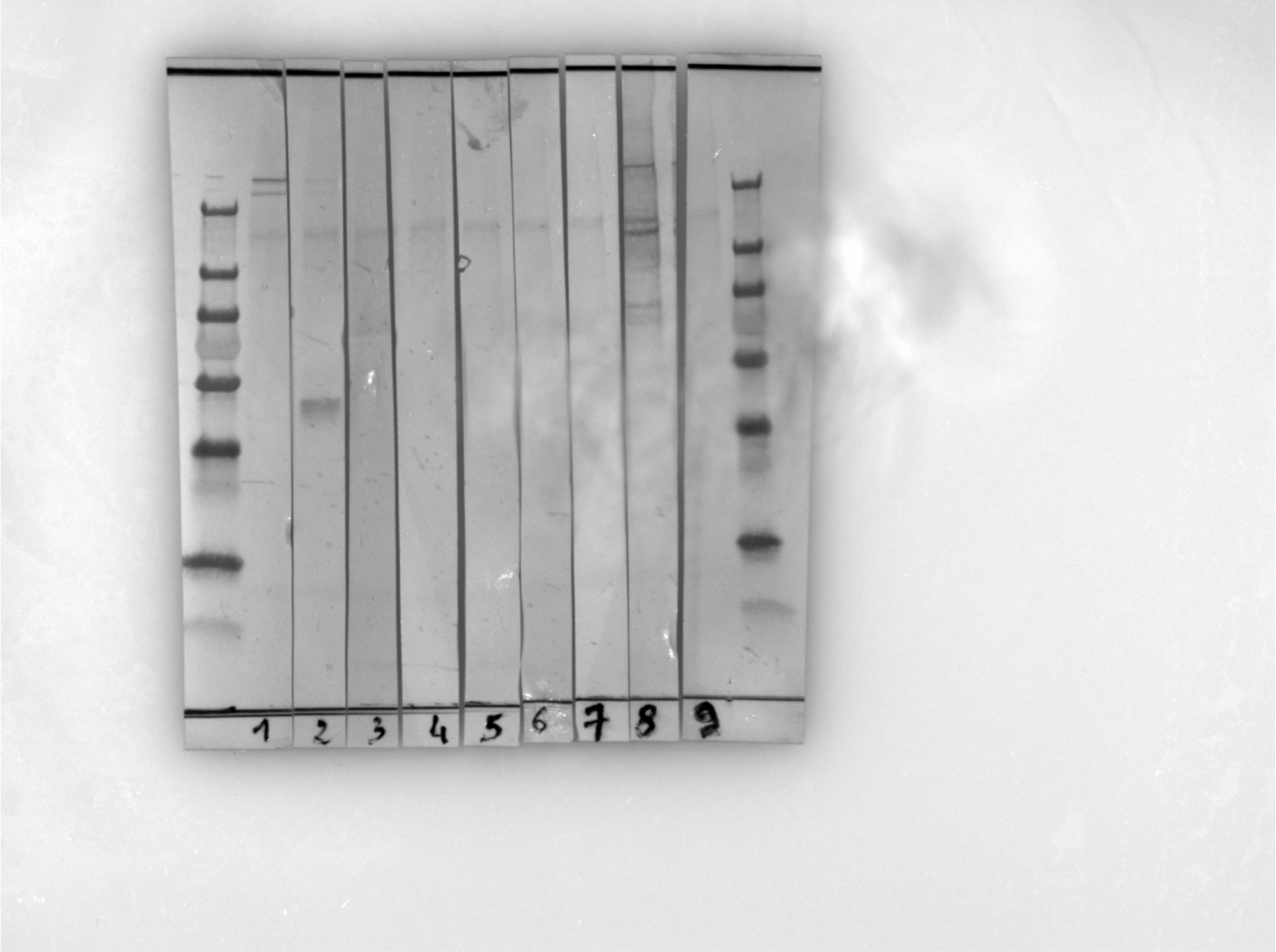

Supplement: Figure 2—source data 2. [file elife-97865-fig2-data2.zip › Figure 2C Unidentified antigens left panel.tif]

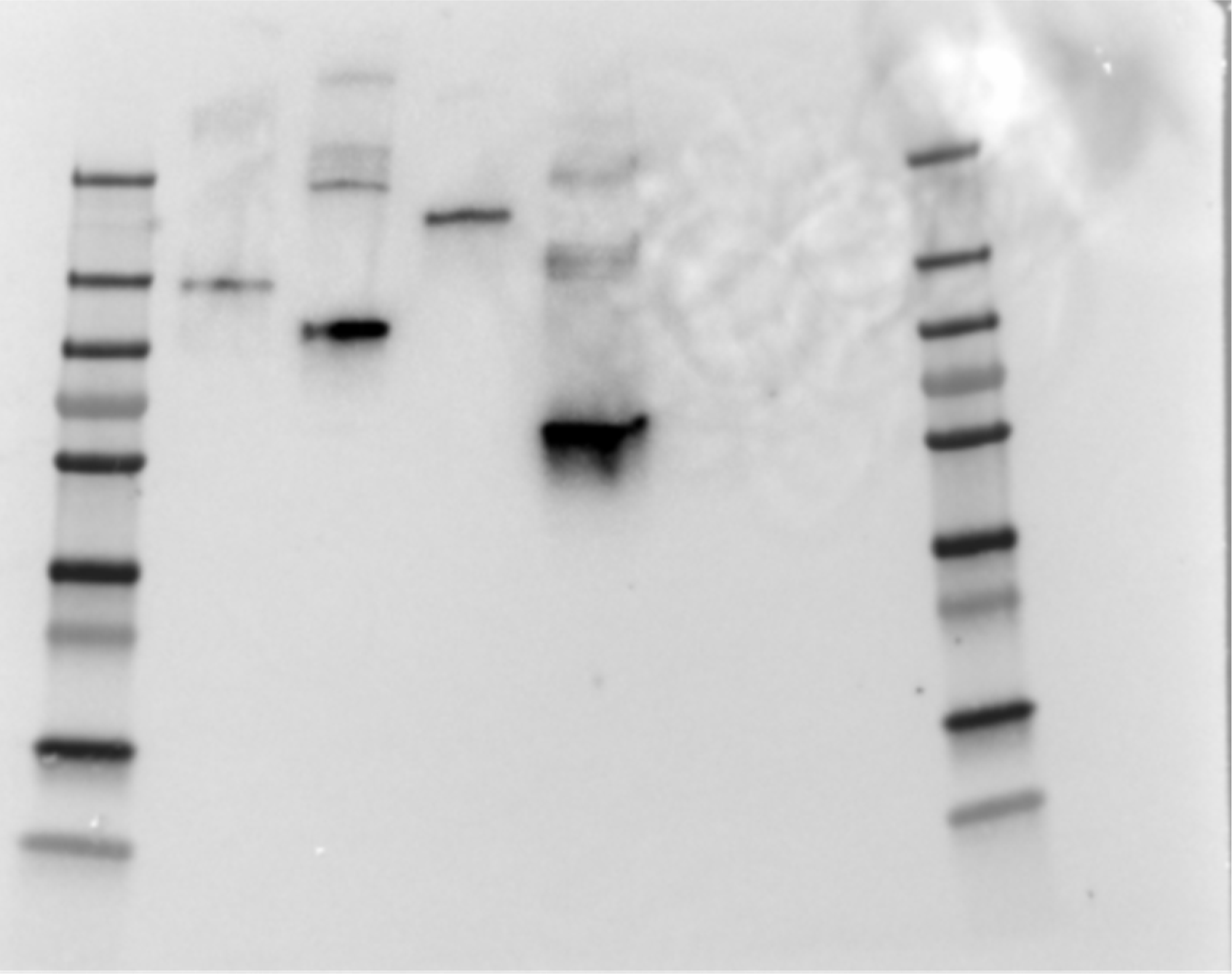

Supplement: Figure 3—source data 2. [file elife-97865-fig3-data2.zip › Figure 3B .tif]
