# Supplementary material for: Target-agnostic identification of human antibodies to Plasmodium falciparum sexual forms reveals cross-stage recognition of glutamate-rich repeats
Source: eLife. 2025 Jan 16;13:RP97865. doi: 10.7554/eLife.97865 (PMC11737873; doi:10.7554/eLife.97865)
Supplement: Supplementary file 1. [file elife-97865-supp1.docx]

**Supplementary file 1. Genetic characteristics of isolated antibodies.** Sequences were analyzed using the IMGT database. Lambda-antibodies are listed over a yellow background and kappa-antibodies over a blue background. V region identity is indicated as a percentage of nucleotides. Antibodies that differ from one another solely through their light chain are indicated using dashed lines (B1C5L/K, B1C8L/K and B1D3L/K).

|  | Heavy Chain | | | | Light Chain | | | |
| --- | --- | --- | --- | --- | --- | --- | --- | --- |
| mAb | V gene | J gene | CDR3 length | V region identity % | V gene | J gene | CDR3 length | V region identity % |
| B1C5L | IGHV1-8*01 | IGHJ5*02 | 16 | 93.06% | IGLV1-44*01 | IGLJ3*02 | 11 | 95.44% |
| B1C5K | IGHV1-8*01 | IGHJ5*02 | 16 | 93.06% | IGKV1-5*03 | IGKJ2*02 | 9 | 89.73% |
| B2C10L | IGHV1-2*02 | IGHJ5*02 | 11 | 93.09% | IGLV2-11*01 | IGLJ1*01 | 9 | 96.53% |
| B2E9L | IGHV3-33*01 | IGHJ4*02 | 17 | 96.53% | IGLV1-51*02 | IGLJ2*01 | 10 | 95.79% |
| B1C8L | IGHV4-61*02 | IGHJ5*02 | 11 | 80.70% | IGLV1-51*02 | IGLJ2*01 | 11 | 86.67% |
| B1C8K | IGHV4-61*02 | IGHJ5*02 | 11 | 80.70% | IGKV3-15*01 | IGKJ1*01 | 10 | 96.42% |
| B1D3L | IGHV1-18*01 | IGHJ6*03 F | 21 | 91.67% | IGLV1-47*01 | IGLJ3*02 | 10 | 88.41% |
| B1D3K | IGHV1-18*01 | IGHJ6*03 F | 21 | 91.67% | IGKV2D-29*01 | IGKJ1*01 | 9 | 97.62% |
| B1F9K | IGHV4-4*07 | IGHJ2*01 | 14 | 58.22% | IGKV1-12*01 | IGKJ3*01 | 9 | 100% |
| B1E7K | IGHV3-33*01 | IGHJ4*02 | 17 | 95.14% | IGKV3-11*01 | IGKJ2*04 | 10 | 97.47% |
| B1C3L | IGHV4-4*07 | IGHJ5*02 | 17 | 100.00% | IGLV2-14*01 | IGLJ2*01 | 10 | 90% |
| B2D10L | IGHV4-34*03 | IGHJ5*02 | 22 | 85.61% | IGLV3-1*01 | IGLJ2*01 | 10 | 96.06% |
| B2F7L | IGHV3-74*03 | IGHJ4*02 | 19 | 85.42% | IGLV2-8*01 | IGLJ1*01 | 10 | 100% |
| B1E11K | IGHV3-7*01 | IGHJ4*01 | 9 | 90.74% | IGKV3-20*01 | IGKJ2*04 | 9 | 90.20% |
