# Supplementary material for: Target-agnostic identification of human antibodies to Plasmodium falciparum sexual forms reveals cross-stage recognition of glutamate-rich repeats
Source: eLife. 2025 Jan 16;13:RP97865. doi: 10.7554/eLife.97865 (PMC11737873; doi:10.7554/eLife.97865)
Supplement: Supplementary file 3. [file elife-97865-supp3.pdf]

Full wwPDB X-ray Structure Validation Report (i)

Nov 1, 2023 – 11:43 AM EDT

PDB ID	:	8US8
Title	:	Crystal structure of B1E11K malarial antibody in complex with RESA repeat peptide
Deposited on	:	2023-10-27
Resolution	:	2.56 Å(reported)

This wwPDB validation report is for manuscript review

This is a Full wwPDB X-ray Structure Validation Report.

This report is produced by the wwPDB biocuration pipeline after annotation of the structure.

We welcome your comments at *validation@mail.wwpdb.org*
A user guide is available at
https://www.wwpdb.org/validation/2017/XrayValidationReportHelp
with specific help available everywhere you see the (i) symbol.

The types of validation reports are described at
http://www.wwpdb.org/validation/2017/FAQs#types.

For Manuscript Review

The following versions of software and data (see references (i)) were used in the production of this report:

MolProbity	:	4.02b-467
Mogul	:	1.8.5 (274361), CSD as541be (2020)
Xtriage (Phenix)	:	1.13
EDS	:	2.36
Percentile statistics	:	20191225.v01 (using entries in the PDB archive December 25th 2019)
Refmac	:	5.8.0158
CCP4	:	7.0.044 (Gargrove)
Ideal geometry (proteins)	:	Engh & Huber (2001)
Ideal geometry (DNA, RNA)	:	Parkinson et al. (1996)

1 Overall quality at a glance (i)

The following experimental techniques were used to determine the structure:
X-RAY DIFFRACTION

The reported resolution of this entry is 2.56 Å.

Percentile scores (ranging between 0-100) for global validation metrics of the entry are shown in the following graphic. The table shows the number of entries on which the scores are based.

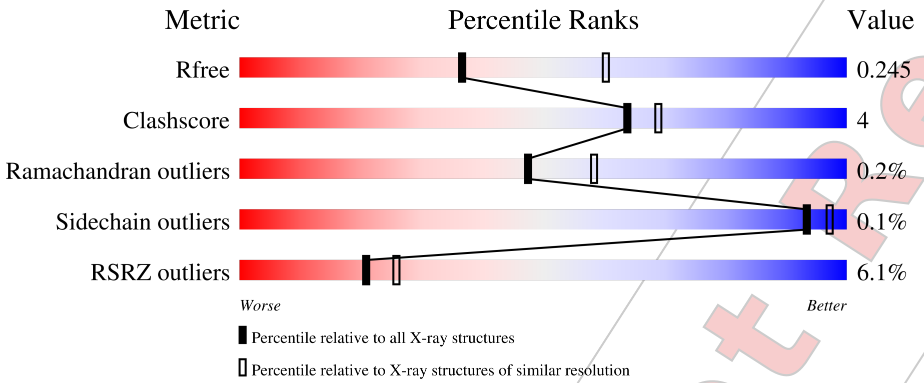

Metric	Whole archive (#Entries)	Similar resolution (#Entries, resolution range(Å))
R_{free}	130704	1279 (2.58-2.54)
Clashscore	141614	1327 (2.58-2.54)
Ramachandran outliers	138981	1312 (2.58-2.54)
Sidechain outliers	138945	1312 (2.58-2.54)
RSRZ outliers	127900	1269 (2.58-2.54)

The table below summarises the geometric issues observed across the polymeric chains and their fit to the electron density. The red, orange, yellow and green segments of the lower bar indicate the fraction of residues that contain outliers for >=3, 2, 1 and 0 types of geometric quality criteria respectively. A grey segment represents the fraction of residues that are not modelled. The numeric value for each fraction is indicated below the corresponding segment, with a dot representing fractions <=5% The upper red bar (where present) indicates the fraction of residues that have poor fit to the electron density. The numeric value is given above the bar.

Mol	Chain	Length	Quality of chain
1	A	219	5% / 89% 6% •
1	H	219	5% / 84% 13% •
2	B	215	8% / 87% 13%
2	L	215	6% / 87% 12% •

Continued on next page...

For Manuscript Review

Continued from previous page...

Mol	Chain	Length	Quality of chain
3	R	19	95% 5%

For Manuscript Review

2 Entry composition (i)

There are 6 unique types of molecules in this entry. The entry contains 6781 atoms, of which 0 are hydrogens and 0 are deuteriums.

In the tables below, the ZeroOcc column contains the number of atoms modelled with zero occupancy, the AltConf column contains the number of residues with at least one atom in alternate conformation and the Trace column contains the number of residues modelled with at most 2 atoms.

- Molecule 1 is a protein called B1E11K Fab A Heavy Chain.

Mol	Chain	Residues	Atoms					ZeroOcc	AltConf	Trace
1	H	211	Total 1591	C 1002	N 275	O 307	S 7	0	0	0
1	A	210	Total 1587	C 1000	N 274	O 306	S 7	0	0	0

- Molecule 2 is a protein called B1E11K Fab A Kappa Light Chain.

Mol	Chain	Residues	Atoms					ZeroOcc	AltConf	Trace
2	L	213	Total 1648	C 1033	N 287	O 324	S 4	0	0	0
2	B	214	Total 1657	C 1038	N 288	O 327	S 4	0	0	0

- Molecule 3 is a protein called Ring-infected erythrocyte surface antigen peptide.

Mol	Chain	Residues	Atoms				ZeroOcc	AltConf	Trace
3	R	18	Total 144	C 84	N 22	O 38	0	0	0

There are 3 discrepancies between the modelled and reference sequences:

Chain	Residue	Modelled	Actual	Comment	Reference
R	0	ACA	-	expression tag	UNP P13830
R	17	GLY	-	expression tag	UNP P13830
R	18	GLY	-	expression tag	UNP P13830

- Molecule 4 is SULFATE ION (three-letter code: SO4) (formula: O_4S).

For Manuscript Review

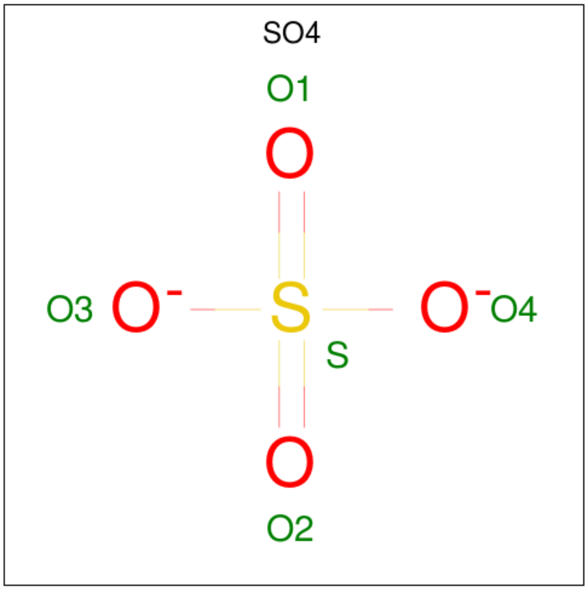

Mol	Chain	Residues	Atoms	ZeroOcc	AltConf
4	L	1	Total O S<br>5 4 1	0	0
4	A	1	Total O S<br>5 4 1	0	0
4	B	1	Total O S<br>5 4 1	0	0
4	R	1	Total O S<br>5 4 1	0	0

- Molecule 5 is 1,2-ETHANEDIOL (three-letter code: EDO) (formula: $C_2H_6O_2$).

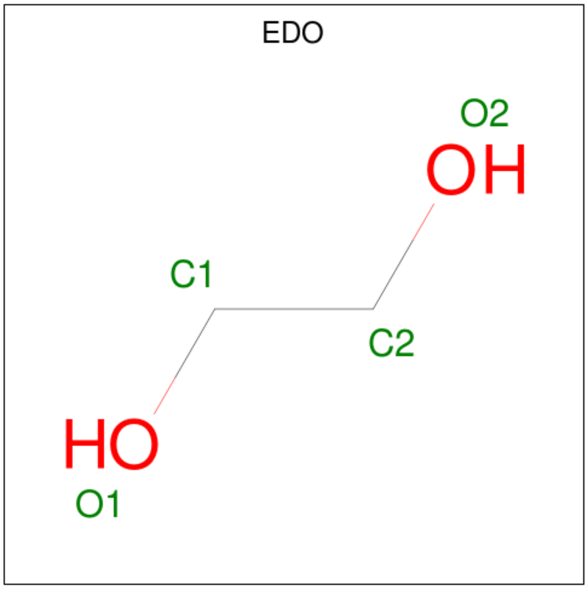

For Manuscript Review

Mol	Chain	Residues	Atoms	ZeroOcc	AltConf
5	L	1	Total C O<br>4 2 2	0	0

- Molecule 6 is water.

Mol	Chain	Residues	Atoms	ZeroOcc	AltConf
6	H	42	Total O<br>42 42	0	0
6	L	21	Total O<br>21 21	0	0
6	A	35	Total O<br>35 35	0	0
6	B	24	Total O<br>24 24	0	0
6	R	8	Total O<br>8 8	0	0

For Manuscript Review

3 Residue-property plots (i)

These plots are drawn for all protein, RNA, DNA and oligosaccharide chains in the entry. The first graphic for a chain summarises the proportions of the various outlier classes displayed in the second graphic. The second graphic shows the sequence view annotated by issues in geometry and electron density. Residues are color-coded according to the number of geometric quality criteria for which they contain at least one outlier: green = 0, yellow = 1, orange = 2 and red = 3 or more. A red dot above a residue indicates a poor fit to the electron density (RSRZ > 2). Stretches of 2 or more consecutive residues without any outlier are shown as a green connector. Residues present in the sample, but not in the model, are shown in grey.

• Molecule 1: B1E11K Fab A Heavy Chain

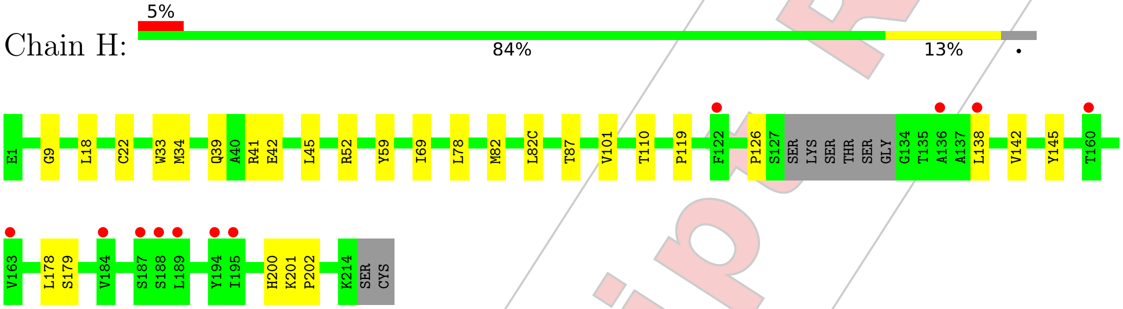

• Molecule 1: B1E11K Fab A Heavy Chain

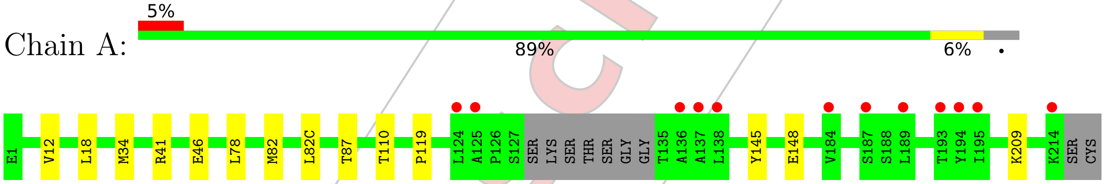

• Molecule 2: B1E11K Fab A Kappa Light Chain

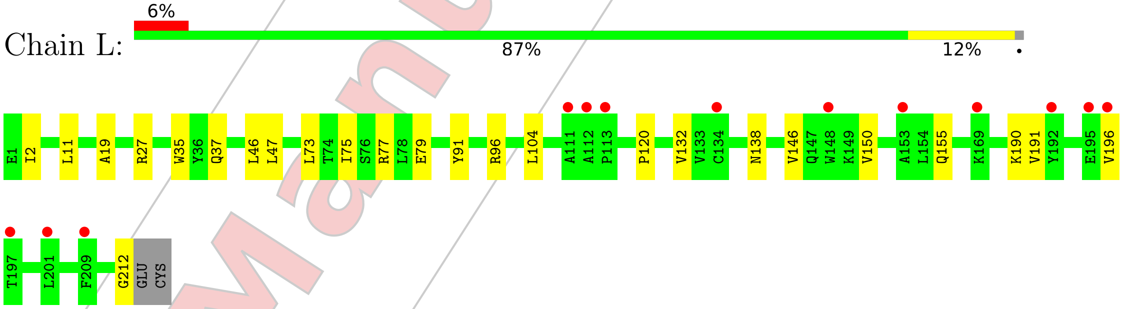

• Molecule 2: B1E11K Fab A Kappa Light Chain

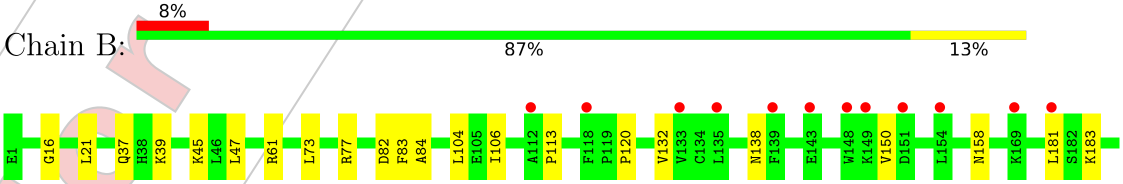

For Manuscript Review

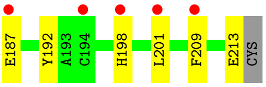

• Molecule 3: Ring-infected erythrocyte surface antigen peptide

Chain R:

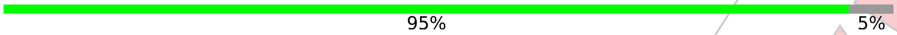

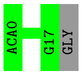

For Manuscript Review

4 Data and refinement statistics (i)

Property	Value	Source
Space group	C 2 2 21	Depositor
Cell constants<br>a, b, c, α, β, γ	78.70Å 186.29Å 131.52Å<br>90.00° 90.00° 90.00°	Depositor
Resolution (Å)	39.67 – 2.56<br>39.67 – 2.56	Depositor<br>EDS
% Data completeness<br>(in resolution range)	99.8 (39.67-2.56)<br>99.8 (39.67-2.56)	Depositor<br>EDS
R_{merge}	(Not available)	Depositor
R_{sym}	(Not available)	Depositor
$< I/\sigma(I) >$ [1]	1.35 (at 2.54Å)	Xtriage
Refinement program	PHENIX 1.20.1_4487	Depositor
R, R_{free}	0.216 , 0.248<br>0.215 , 0.245	Depositor<br>DCC
R_{free} test set	1576 reflections (5.00%)	wwPDB-VP
Wilson B-factor (Å^2)	74.9	Xtriage
Anisotropy	0.135	Xtriage
Bulk solvent k_{sol}(e/Å^3), B_{sol}(Å^2)	0.31 , 53.9	EDS
L-test for twinning[2]	$< \|L\| > = 0.49$, $< L^2 > = 0.32$	Xtriage
Estimated twinning fraction	No twinning to report.	Xtriage
F_o,F_c correlation	0.95	EDS
Total number of atoms	6781	wwPDB-VP
Average B, all atoms (Å^2)	89.0	wwPDB-VP

Xtriage's analysis on translational NCS is as follows: *The largest off-origin peak in the Patterson function is 4.61% of the height of the origin peak. No significant pseudotranslation is detected.*

[1]Intensities estimated from amplitudes.

[2]Theoretical values of $< |L| >$, $< L^2 >$ for acentric reflections are 0.5, 0.333 respectively for untwinned datasets, and 0.375, 0.2 for perfectly twinned datasets.

For Manuscript Review

5 Model quality (i)

5.1 Standard geometry (i)

Bond lengths and bond angles in the following residue types are not validated in this section: ACA, EDO, SO4

The Z score for a bond length (or angle) is the number of standard deviations the observed value is removed from the expected value. A bond length (or angle) with $|Z| > 5$ is considered an outlier worth inspection. RMSZ is the root-mean-square of all Z scores of the bond lengths (or angles).

Mol	Chain	Bond lengths		Bond angles	
		RMSZ	#$\|Z\|$ >5	RMSZ	#$\|Z\|$ >5
1	A	0.25	0/1625	0.49	0/2212
1	H	0.25	0/1629	0.49	0/2217
2	B	0.24	0/1695	0.50	0/2301
2	L	0.25	0/1686	0.51	0/2289
3	R	0.24	0/135	0.44	0/182
All	All	0.25	0/6770	0.49	0/9201

There are no bond length outliers.

There are no bond angle outliers.

There are no chirality outliers.

There are no planarity outliers.

5.2 Too-close contacts (i)

In the following table, the Non-H and H(model) columns list the number of non-hydrogen atoms and hydrogen atoms in the chain respectively. The H(added) column lists the number of hydrogen atoms added and optimized by MolProbity. The Clashes column lists the number of clashes within the asymmetric unit, whereas Symm-Clashes lists symmetry-related clashes.

Mol	Chain	Non-H	H(model)	H(added)	Clashes	Symm-Clashes
1	A	1587	0	1548	9	0
1	H	1591	0	1551	18	0
2	B	1657	0	1621	16	0
2	L	1648	0	1615	13	0
3	R	144	0	122	0	0
4	A	5	0	0	0	0
4	B	5	0	0	0	0
4	L	5	0	0	0	0

Continued on next page...

For Manuscript Review

Continued from previous page...

Mol	Chain	Non-H	H(model)	H(added)	Clashes	Symm-Clashes
4	R	5	0	0	0	0
5	L	4	0	6	0	0
6	A	35	0	0	2	0
6	B	24	0	0	1	0
6	H	42	0	0	0	0
6	L	21	0	0	0	0
6	R	8	0	0	0	0
All	All	6781	0	6463	55	0

The all-atom clashscore is defined as the number of clashes found per 1000 atoms (including hydrogen atoms). The all-atom clashscore for this structure is 4.

All (55) close contacts within the same asymmetric unit are listed below, sorted by their clash magnitude.

Atom-1	Atom-2	Interatomic distance (Å)	Clash overlap (Å)
2:L:77:ARG:NH1	2:L:79:GLU:OE1	2.33	0.61
2:B:83:PHE:CG	2:B:106:ILE:HG12	2.36	0.61
2:L:190:LYS:HA	2:L:212:GLY:HA2	1.83	0.59
1:H:82:MET:HB3	1:H:82(C):LEU:HD21	1.85	0.59
2:L:37:GLN:HB2	2:L:47:LEU:HD11	1.85	0.59
2:B:120:PRO:HD3	2:B:132:VAL:HG22	1.84	0.59
1:A:46:GLU:OE2	6:A:401:HOH:O	2.18	0.57
2:B:37:GLN:HB2	2:B:47:LEU:HD11	1.86	0.57
1:H:34:MET:HB3	1:H:78:LEU:HD22	1.86	0.56
1:H:126:PRO:HG3	1:H:138:LEU:HB3	1.88	0.55
1:A:41:ARG:NH2	1:A:148:GLU:OE2	2.34	0.54
2:B:198:HIS:HB3	2:B:201:LEU:HB2	1.90	0.54
2:B:61:ARG:NE	2:B:82:ASP:OD2	2.40	0.54
2:B:16:GLY:HA2	2:B:77:ARG:HG3	1.91	0.52
1:H:39:GLN:HB2	1:H:45:LEU:HD23	1.90	0.52
1:A:209:LYS:NZ	6:A:404:HOH:O	2.38	0.52
2:L:19:ALA:HB3	2:L:75:ILE:HB	1.93	0.51
1:A:82:MET:HB3	1:A:82(C):LEU:HD21	1.92	0.50
2:L:120:PRO:HD3	2:L:132:VAL:HG22	1.94	0.50
2:B:213:GLU:N	2:B:213:GLU:OE1	2.45	0.50
2:B:150:VAL:HG22	2:B:192:TYR:HD1	1.78	0.49
2:L:190:LYS:HG3	2:L:191:VAL:HG23	1.94	0.48
2:B:158:ASN:HD22	2:B:181:LEU:HD21	1.78	0.48
2:B:150:VAL:HG22	2:B:192:TYR:CD1	2.49	0.48
1:H:87:THR:HG23	1:H:110:THR:HA	1.96	0.47
1:A:119:PRO:HB3	1:A:145:TYR:HB3	1.97	0.47

Continued on next page...

For Manuscript Review

Continued from previous page...

Atom-1	Atom-2	Interatomic distance (Å)	Clash overlap (Å)
1:H:9:GLY:HA2	1:H:18:LEU:HD13	1.95	0.47
1:H:22:CYS:HB3	1:H:78:LEU:HB3	1.97	0.47
1:H:142:VAL:HB	1:H:178:LEU:HB3	1.98	0.46
2:L:150:VAL:HG23	2:L:155:GLN:HG3	1.97	0.46
1:A:12:VAL:HG11	1:A:82(C):LEU:HD13	1.97	0.46
1:H:119:PRO:HB3	1:H:145:TYR:HB3	1.98	0.46
1:H:41:ARG:HG2	1:H:42:GLU:HG3	1.97	0.45
1:A:87:THR:HG23	1:A:110:THR:HA	1.99	0.44
2:L:91:TYR:HA	2:L:96:ARG:HB3	2.00	0.43
2:L:146:VAL:HG12	2:L:196:VAL:HG22	2.00	0.43
1:H:201:LYS:N	1:H:202:PRO:HD2	2.34	0.43
2:B:113:PRO:HD2	2:B:201:LEU:HD13	2.00	0.43
1:H:101:VAL:HG22	2:L:46:LEU:HD23	2.01	0.43
2:B:21:LEU:HD12	2:B:73:LEU:HD23	2.01	0.42
1:H:33:TRP:CZ2	1:H:52:ARG:HG3	2.54	0.42
2:L:11:LEU:HD23	2:L:104:LEU:HD13	2.01	0.42
1:H:200:HIS:NE2	1:H:202:PRO:HG2	2.34	0.42
1:A:34:MET:HB3	1:A:78:LEU:HD22	2.02	0.42
1:H:178:LEU:HD12	1:H:179:SER:H	1.85	0.41
2:B:45:LYS:NZ	6:B:405:HOH:O	2.42	0.41
1:H:59:TYR:CZ	1:H:69:ILE:HG22	2.55	0.41
2:B:39:LYS:HG2	2:B:84:ALA:HB2	2.01	0.41
1:H:142:VAL:N	1:H:178:LEU:O	2.53	0.41
2:L:2:ILE:HD11	2:L:27:ARG:HH11	1.86	0.41
2:B:192:TYR:HB2	2:B:209:PHE:CE1	2.56	0.41
2:B:183:LYS:O	2:B:187:GLU:HG3	2.21	0.40
1:A:18:LEU:HD12	1:A:18:LEU:HA	1.93	0.40
1:H:33:TRP:CE2	1:H:52:ARG:HG3	2.57	0.40
2:L:35:TRP:CE2	2:L:73:LEU:HB2	2.56	0.40

There are no symmetry-related clashes.

5.3 Torsion angles (i)

5.3.1 Protein backbone (i)

In the following table, the Percentiles column shows the percent Ramachandran outliers of the chain as a percentile score with respect to all X-ray entries followed by that with respect to entries of similar resolution.

The Analysed column shows the number of residues for which the backbone conformation was analysed, and the total number of residues.

For Manuscript Review

Mol	Chain	Analysed	Favoured	Allowed	Outliers	Percentiles	
1	A	206/219 (94%)	203 (98%)	3 (2%)	0	100	100
1	H	207/219 (94%)	203 (98%)	4 (2%)	0	100	100
2	B	212/215 (99%)	197 (93%)	14 (7%)	1 (0%)	29	39
2	L	211/215 (98%)	199 (94%)	11 (5%)	1 (0%)	29	39
3	R	15/19 (79%)	15 (100%)	0	0	100	100
All	All	851/887 (96%)	817 (96%)	32 (4%)	2 (0%)	47	58

All (2) Ramachandran outliers are listed below:

Mol	Chain	Res	Type
2	L	138	ASN
2	B	138	ASN

5.3.2 Protein sidechains (i)

In the following table, the Percentiles column shows the percent sidechain outliers of the chain as a percentile score with respect to all X-ray entries followed by that with respect to entries of similar resolution.

The Analysed column shows the number of residues for which the sidechain conformation was analysed, and the total number of residues.

Mol	Chain	Analysed	Rotameric	Outliers	Percentiles	
1	A	176/183 (96%)	176 (100%)	0	100	100
1	H	176/183 (96%)	176 (100%)	0	100	100
2	B	186/187 (100%)	185 (100%)	1 (0%)	88	93
2	L	185/187 (99%)	185 (100%)	0	100	100
3	R	16/16 (100%)	16 (100%)	0	100	100
All	All	739/756 (98%)	738 (100%)	1 (0%)	93	97

All (1) residues with a non-rotameric sidechain are listed below:

Mol	Chain	Res	Type
2	B	104	LEU

Sometimes sidechains can be flipped to improve hydrogen bonding and reduce clashes. There are no such sidechains identified.

For Manuscript Review

5.3.3 RNA (i)

There are no RNA molecules in this entry.

5.4 Non-standard residues in protein, DNA, RNA chains (i)

1 non-standard protein/DNA/RNA residue is modelled in this entry.

In the following table, the Counts columns list the number of bonds (or angles) for which Mogul statistics could be retrieved, the number of bonds (or angles) that are observed in the model and the number of bonds (or angles) that are defined in the Chemical Component Dictionary. The Link column lists molecule types, if any, to which the group is linked. The Z score for a bond length (or angle) is the number of standard deviations the observed value is removed from the expected value. A bond length (or angle) with $|Z| > 2$ is considered an outlier worth inspection. RMSZ is the root-mean-square of all Z scores of the bond lengths (or angles).

Mol	Type	Chain	Res	Link	Bond lengths			Bond angles		
					Counts	RMSZ	$\#\|Z\| > 2$	Counts	RMSZ	$\#\|Z\| > 2$
3	ACA	R	0	3	7,7,8	0.55	0	6,6,8	0.91	0

In the following table, the Chirals column lists the number of chiral outliers, the number of chiral centers analysed, the number of these observed in the model and the number defined in the Chemical Component Dictionary. Similar counts are reported in the Torsion and Rings columns. '-' means no outliers of that kind were identified.

Mol	Type	Chain	Res	Link	Chirals	Torsions	Rings
3	ACA	R	0	3	-	0/4/5/6	-

There are no bond length outliers.

There are no bond angle outliers.

There are no chirality outliers.

There are no torsion outliers.

There are no ring outliers.

No monomer is involved in short contacts.

5.5 Carbohydrates (i)

There are no monosaccharides in this entry.

For Manuscript Review

5.6 Ligand geometry (i)

5 ligands are modelled in this entry.

In the following table, the Counts columns list the number of bonds (or angles) for which Mogul statistics could be retrieved, the number of bonds (or angles) that are observed in the model and the number of bonds (or angles) that are defined in the Chemical Component Dictionary. The Link column lists molecule types, if any, to which the group is linked. The Z score for a bond length (or angle) is the number of standard deviations the observed value is removed from the expected value. A bond length (or angle) with $|Z| > 2$ is considered an outlier worth inspection. RMSZ is the root-mean-square of all Z scores of the bond lengths (or angles).

Mol	Type	Chain	Res	Link	Bond lengths			Bond angles		
					Counts	RMSZ	$\#\|Z\| > 2$	Counts	RMSZ	$\#\|Z\| > 2$
5	EDO	L	302	-	3,3,3	0.54	0	2,2,2	0.05	0
4	SO4	L	301	-	4,4,4	0.14	0	6,6,6	0.05	0
4	SO4	R	101	-	4,4,4	0.15	0	6,6,6	0.05	0
4	SO4	B	301	-	4,4,4	0.15	0	6,6,6	0.04	0
4	SO4	A	301	-	4,4,4	0.14	0	6,6,6	0.07	0

In the following table, the Chirals column lists the number of chiral outliers, the number of chiral centers analysed, the number of these observed in the model and the number defined in the Chemical Component Dictionary. Similar counts are reported in the Torsion and Rings columns. '-' means no outliers of that kind were identified.

Mol	Type	Chain	Res	Link	Chirals	Torsions	Rings
5	EDO	L	302	-	-	0/1/1/1	-

There are no bond length outliers.

There are no bond angle outliers.

There are no chirality outliers.

There are no torsion outliers.

There are no ring outliers.

No monomer is involved in short contacts.

5.7 Other polymers (i)

There are no such residues in this entry.

5.8 Polymer linkage issues (i)

There are no chain breaks in this entry.

For Manuscript Review

6 Fit of model and data (i)

6.1 Protein, DNA and RNA chains (i)

In the following table, the column labelled '#RSRZ> 2' contains the number (and percentage) of RSRZ outliers, followed by percent RSRZ outliers for the chain as percentile scores relative to all X-ray entries and entries of similar resolution. The OWAB column contains the minimum, median, 95th percentile and maximum values of the occupancy-weighted average B-factor per residue. The column labelled 'Q< 0.9' lists the number of (and percentage) of residues with an average occupancy less than 0.9.

Mol	Chain	Analysed	<RSRZ>	#RSRZ>2			OWAB(Å^2)	Q<0.9
1	A	210/219 (95%)	0.24	12 (5%)	23	30	43, 71, 142, 164	0
1	H	211/219 (96%)	0.34	11 (5%)	27	34	39, 74, 143, 163	0
2	B	214/215 (99%)	0.62	17 (7%)	12	17	43, 100, 154, 179	0
2	L	213/215 (99%)	0.43	13 (6%)	21	26	49, 97, 160, 184	0
3	R	17/19 (89%)	0.17	0	100	100	50, 64, 104, 111	0
All	All	865/887 (97%)	0.40	53 (6%)	21	26	39, 82, 150, 184	0

All (53) RSRZ outliers are listed below:

Mol	Chain	Res	Type	RSRZ
1	H	184	VAL	4.9
1	H	194	TYR	4.4
1	A	138	LEU	4.1
2	B	148	TRP	4.1
2	L	112	ALA	4.0
2	B	169	LYS	3.8
1	H	163	VAL	3.6
2	B	201	LEU	3.5
1	H	138	LEU	3.4
2	L	196	VAL	3.4
1	H	189	LEU	3.3
2	B	118	PHE	3.2
2	L	169	LYS	3.2
2	L	113	PRO	3.1
1	A	184	VAL	3.1
1	H	195	ILE	3.0
1	H	136	ALA	2.9
2	B	149	LYS	2.9
2	B	209	PHE	2.9
2	B	135	LEU	2.9

Continued on next page...

For Manuscript Review

Continued from previous page...

Mol	Chain	Res	Type	RSRZ
2	L	197	THR	2.8
1	A	194	TYR	2.8
2	L	192	TYR	2.7
2	L	148	TRP	2.7
1	H	188	SER	2.7
1	A	125	ALA	2.7
1	A	137	ALA	2.6
2	B	194	CYS	2.6
1	H	160	THR	2.5
2	L	153	ALA	2.5
1	A	189	LEU	2.5
2	B	151	ASP	2.5
2	B	143	GLU	2.5
2	B	112	ALA	2.5
2	L	134	CYS	2.4
2	L	195	GLU	2.4
1	A	195	ILE	2.3
2	L	209	PHE	2.3
2	B	187	GLU	2.3
2	L	111	ALA	2.3
1	H	122	PHE	2.3
2	B	139	PHE	2.3
2	B	181	LEU	2.3
1	A	187	SER	2.2
2	B	133	VAL	2.2
1	H	187	SER	2.2
2	B	198	HIS	2.2
1	A	214	LYS	2.2
1	A	124	LEU	2.2
1	A	193	THR	2.1
2	B	154	LEU	2.1
1	A	136	ALA	2.0
2	L	201	LEU	2.0

6.2 Non-standard residues in protein, DNA, RNA chains (i)

In the following table, the Atoms column lists the number of modelled atoms in the group and the number defined in the chemical component dictionary. The B-factors column lists the minimum, median, 95th percentile and maximum values of B factors of atoms in the group. The column labelled 'Q< 0.9' lists the number of atoms with occupancy less than 0.9.

For Manuscript Review

Mol	Type	Chain	Res	Atoms	RSCC	RSR	B-factors(Å^2)	Q<0.9
3	ACA	R	0	8/9	0.84	0.20	72,76,80,83	0

6.3 Carbohydrates (i)

There are no monosaccharides in this entry.

6.4 Ligands (i)

In the following table, the Atoms column lists the number of modelled atoms in the group and the number defined in the chemical component dictionary. The B-factors column lists the minimum, median, 95th percentile and maximum values of B factors of atoms in the group. The column labelled 'Q< 0.9' lists the number of atoms with occupancy less than 0.9.

Mol	Type	Chain	Res	Atoms	RSCC	RSR	B-factors(Å^2)	Q<0.9
5	EDO	L	302	4/4	0.86	0.25	85,85,85,85	0
4	SO4	A	301	5/5	0.87	0.25	62,62,62,62	5
4	SO4	R	101	5/5	0.94	0.23	58,58,58,58	5
4	SO4	L	301	5/5	0.95	0.16	94,96,101,107	0
4	SO4	B	301	5/5	0.95	0.13	109,109,109,109	0

6.5 Other polymers (i)

There are no such residues in this entry.

For Manuscript Review
