# Supplementary material for: Target-agnostic identification of human antibodies to Plasmodium falciparum sexual forms reveals cross-stage recognition of glutamate-rich repeats
Source: eLife. 2025 Jan 16;13:RP97865. doi: 10.7554/eLife.97865 (PMC11737873; doi:10.7554/eLife.97865)
Supplement: Supplementary file 4. [file elife-97865-supp4.docx]

**Supplementary file 4. Electrostatic Interactions between B1E11K Fabs and RESA P2 (16AA) peptide.**

| **RESA P2** | **Fab A Heavy** | **Fab A Kappa** | **Fab B Heavy** | **Fab B Kappa** |
| --- | --- | --- | --- | --- |
| **E1*** |  | **H49*** |  |  |
| **E2*** | **R94*** |  |  |  |
| **E2 [O]** | **S96 [N]** |  |  |  |
| **E2 [O]** | **G97 [N]** |  |  |  |
| **N3 [Nδ2]** | **S31 [O]** |  |  |  |
| **N3 [Oδ1]** | **W33 [N]** |  |  |  |
| **V4 [N]** | **S96 [Oγ]** |  |  |  |
| **V4 [O]** | **S96 [Oγ]** |  |  |  |
| **V4 [O]** |  | **R96 [Nη]** |  |  |
| **E5 [O]** |  | **R96 [Nη]** |  |  |
| **E5 [Oε]** |  |  |  | **G57 [N]** |
| **E6*** |  | **R96*** |  |  |
| **E6 [Oε]** |  | **S94 [N]** |  |  |
| **E6 [O]** |  |  |  | **H49 [Nε2]** |
| **N7 [N]** |  | **G92 [O]** |  |  |
| **N7 [Nδ2]** |  | **G92 [O]** |  |  |
| **E9*** |  | **R93*** |  |  |
| **E10*** |  |  | **R94*** |  |
| **E10 [O]** |  |  | **S96 [N]** |  |
| **E10 [O]** |  |  | **G97 [N]** |  |
| **N11 [Nδ2]** |  |  | **S31 [O]** |  |
| **N11[Oδ1]** |  |  | **W33 [N]** |  |
| **V12 [N]** |  |  | **S96 [Oγ]** |  |
| **V12 [O]** |  |  | **S96 [Oγ]** |  |
| **V12 [O]** |  |  |  | **R96 [Nη]** |
| **E13*** |  |  | **R52*** |  |
| **E13 [Oε]** |  |  | **W33 [Nε1]** |  |
| **E14*** |  |  |  | **R96*** |
| **E14 [Oε]** |  |  |  | **S94 [N]** |
| **E14 [Oε]** |  |  |  | **S94 [Oγ]** |
| **N15 [N]** |  |  |  | **G92 [O]** |
| **N15 [Nδ2]** |  |  |  | **G92 [O]** |

The asterisk (*) denotes a salt bridge. Atoms involved in hydrogen bonding interactions are shown in square brackets.
