# Supplementary material for: Target-agnostic identification of human antibodies to Plasmodium falciparum sexual forms reveals cross-stage recognition of glutamate-rich repeats
Source: eLife. 2025 Jan 16;13:RP97865. doi: 10.7554/eLife.97865 (PMC11737873; doi:10.7554/eLife.97865)
Supplement: Supplementary file 5. [file elife-97865-supp5.docx]

**Supplementary file 5. Electrostatic Homotypic Interactions between B1E11K Fab A and Fab B**

| **Fab A Heavy** | **Fab A Kappa** | **Fab B Heavy** | **Fab B Kappa** |
| --- | --- | --- | --- |
| **R52*** |  |  | **D60* (FR)** |
| **H52A*** |  |  | **D60* (FR)** |
| **S55 [Oγ]** |  |  | **R54 [Nη1]** |
| **E56 [Oε]** |  |  | **R54 [N]** |
| **E56*** |  |  | **R54*** |
| **Y58 [Oη]** |  |  | **S53 [Oγ]** |
|  | **T29 [O]** | **Y32 [Oη]** |  |
|  | **R27 [Nη]** | **S31 [O]** |  |

The asterisk (*) denotes a salt bridge. Atoms involved in hydrogen bonding interactions are shown in square brackets.
